# Coverage Planning for UVC Irradiation: Robot Surface Disinfection Based on Swarm Intelligence Algorithm

**DOI:** 10.3390/s24113418

**Published:** 2024-05-26

**Authors:** Peiyao Guo, Dekun Luo, Yizhen Wu, Sheng He, Jianyu Deng, Huilu Yao, Wenhong Sun, Jicai Zhang

**Affiliations:** 1 Research Center for Optoelectronic Materials and Devices, Guangxi Key Laboratory for the Relativistic Astrophysics, School of Physical Science & Technology, Guangxi University, Nanning 530004, China; peiyaoguo126@126.com (P.G.); 2207301092@st.gxu.edu.cn (D.L.); 2107301146@st.gxu.edu.cn (Y.W.); hscestlavie@163.com (S.H.); ddjy2000@gmail.com (J.D.); 2 School of Electrical Engineering, Guangxi University, Nanning 530004, China; yhl@gxu.edu.cn; 3 MOE Key Laboratory of New Processing Technology for Nonferrous Metals and Materials, Guangxi Key Laboratory of Processing for Non-Ferrous Metals and Featured Materials, Nanning 530004, China; 4 Third Generation Semiconductor Industry Research Institute, Guangxi University, Nanning 530004, China; 5 College of Mathematics and Physics, Beijing University of Chemical Technology, Beijing 100029, China; jczhang@mail.buct.edu.cn

**Keywords:** disinfection robot, irradiation planner, coverage path planning, AIGaN-based UVC LED

## Abstract

Ultraviolet (UV) radiation has been widely utilized as a disinfection strategy to effectively eliminate various pathogens. The disinfection task achieves complete coverage of object surfaces by planning the motion trajectory of autonomous mobile robots and the UVC irradiation strategy. This introduces an additional layer of complexity to path planning, as every point on the surface of the object must receive a certain dose of irradiation. Nevertheless, the considerable dosage required for virus inactivation often leads to substantial energy consumption and dose redundancy in disinfection tasks, presenting challenges for the implementation of robots in large-scale environments. Optimizing energy consumption of light sources has become a primary concern in disinfection planning, particularly in large-scale settings. Addressing the inefficiencies associated with dosage redundancy, this study proposes a dose coverage planning framework, utilizing MOPSO to solve the multi-objective optimization model for planning UVC dose coverage. Diverging from conventional path planning methodologies, our approach prioritizes the intrinsic characteristics of dose accumulation, integrating a UVC light efficiency factor to mitigate dose redundancy with the aim of reducing energy expenditure and enhancing the efficiency of robotic disinfection. Empirical trials conducted with autonomous disinfecting robots in real-world settings have corroborated the efficacy of this model in deactivating viruses.

## 1. Introduction

The global dissemination of respiratory diseases underscores the vital importance of regular surface disinfection in mitigating the spread of infectious pathogens [1]. Ultraviolet irradiation technology, particularly within the UVC spectrum (200 nm to 280 nm), emerges as a highly promising approach for surface disinfection owing to its potent antimicrobial properties [2]. Extensive testing has demonstrated the efficacy of UV irradiation in effectively deactivating various pathogens, including coronaviruses, bacteria, and protozoa [3]. Utilization of UVC devices has been proven to significantly reduce microbial proliferation, rendering it a viable method for non-contact disinfection. Since the onset of the SARS-CoV-2 pandemic, the effectiveness of UV in environmental disinfection has led to an increased adoption of UV lamps in hospitals and other healthcare facilities [4,5].

According to a study by the University of British Columbia [6], there are two critical factors for pathogen inactivation using deep ultraviolet (UVC) light: sufficient irradiance and accumulation of dose on surfaces to reach the pathogen’s inactivation threshold. Each microorganism has a specific UVC dose required for inactivation, known as the inactivation threshold dose. For instance, inactivating the SARS-CoV-2 virus requires a surface accumulation of a 16.9 mJ/cm^2^ dosage [7]. This paper utilizes high-power UVC LEDs based on AlGaN chips, with each LED emitting stable 273 nm deep ultraviolet light under 500 mA excitation. Experimental tests confirm that a single UVC LED achieves an irradiance of 2.38 W, meeting the instantaneous irradiance requirements. The performance parameters of the UVC LED irradiance are summarized in Section 5.1. Section 3 of this paper discusses the design considerations undertaken to achieve the required irradiance dose.

While UVC disinfection solutions offer convenience, they also pose risks when used in real-world scenarios, including potential harm to human skin, eyes, and other organs if exposed to ultraviolet light for prolonged periods. Moreover, the placement of fixed UV light sources is contentious due to the energy attenuation of UVC light, which diminishes with the square of the distance, resulting in slow disinfection in distant areas [2].

To address these issues, equipping autonomous unmanned ground vehicles (UGVs) with UV emitters seems to be an effective solution, enhancing the efficiency of UVC utilization while ensuring human safety [8,9]. The utilization of automation and robotics has become a trend across various fields, especially in medical and hazardous tasks [10,11]. This not only reduces risks for operators but also significantly enhances task execution efficiency and precision. Autonomous robots offer a low-cost, non-contact disinfection method, minimizing human exposure risks [10,12]. The research community has explored integrating ultraviolet light with autonomous robots [13,14,15,16]. For instance, trajectory planners based on genetic algorithms have been proposed to optimize the disinfection process [17]. These systems pre-calculate disinfection routes offline based on known environmental maps, which are then executed by robots. Other innovations include UV robots equipped with Raspberry Pi for navigation and obstacle avoidance, designed with a remote control scheme for operators [18]; however, this mode does not plan disinfection doses. Additionally, robots with arms have been designed to increase flexibility and irradiation coverage during disinfection mobility, along with maps to determine the projected UVC dose onto environmental surfaces [19].

The robotics domain has made significant contributions to UVC disinfection, yet there is room for improvement, particularly in optimizing energy efficiency and managing the power consumption of UVC light sources. Given the significant disparity in energy consumption between the light sources and the chassis during disinfection tasks, optimizing the energy efficiency of the light sources in expansive environments emerges as a pivotal aspect of disinfection strategy. UVC solutions require high intensity and prolonged exposure to effectively disinfect, posing challenges to the lifespan, effectiveness, and energy consumption of both UVC lamps and the robots carrying them. Insufficient energy may lead to incomplete disinfection tasks, posing risks of secondary infection to personnel. These issues remain to be addressed.

**Figure 1:**
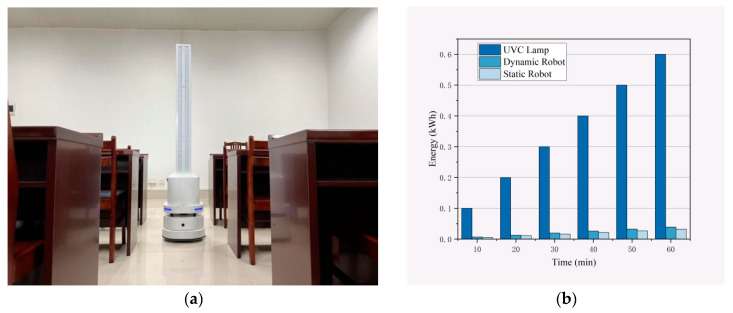
Energy consumption of the robot chassis and UVC lamp set as a function of working time. (**a**) Illustration of irradiation of a robot equipped with UVC lamps. (**b**) As the working time varies, the energy consumption ratio between the UVC lamp group and the robot chassis is observed. It is evident that the UVC lamps account for a significant portion of the energy consumed for disinfection tasks.

This research introduces an irradiation-based disinfection robot system, depicted in Figure 1a, equipped with an array of eight strips, each containing high-power AlGaN-based deep ultraviolet (UVC) LED chips. Each UVC LED strip is rated at 72 W, culminating in a collective rated power of 576 W for the assembly. As demonstrated in Figure 1b, the energy consumption of the UVC light groups substantially exceeds that of the robot chassis across various temporal observations. For instance, a 30 min operation in a confined space consumes approximately 1080 kJ for illumination, contrasted with merely 70.2 kJ for robot movement. This gap widens in larger spaces, where a 60 min task necessitates 2160 kJ for lighting compared to only 140.4 kJ for locomotion. These observations underscore the necessity of focusing on light source energy optimization in large-scale disinfection operations.

In pursuit of enhancing the efficiency of light source utilization, this paper develops a multi-objective optimization model tailored for UVC dose coverage planning. The model employs the Multi-Objective Particle Swarm Optimization (MOPSO) technique to strategize waypoint deployment. Diverging from conventional path optimization strategies that prioritize chassis movement, our approach focuses on maximizing dose accumulation. By integrating a UVC dose efficiency factor, we aim to minimize redundant dosing and optimize energy use, thereby elevating the overall disinfection efficacy of the robot system. The principal contributions of this article are outlined as follows:

- An UVC irradiation dose map has been constructed based on the grid map obtained from SLAM.
- An energy-efficient UVC dose coverage planner (UVC-DCP) has been developed, formulating a multi-objective optimization model for the planning of irradiation tasks. This model focuses on optimizing the deployment of waypoints and static irradiation durations to minimize UVC irradiation energy consumption.
- Considering the complexity of disinfection environments, a full coverage path planning approach utilizing regional segmentation has been adopted.
- The efficacy of the proposed methods has been substantiated through experimental and simulation studies, which also analyze the strategies for inactivating different pathogens.

## 2. Related Work

Autonomous UVC disinfection engages classic robotics problems, akin to comprehensive path and energy coverage planning. Energy coverage planning orchestrates light fields to administer doses on surfaces and optimize UVC usage for efficient disinfection. Path planning computes robot trajectories that allow light coverage across all surfaces, ensuring optimal robot paths while maintaining safe collision distances. The introduced disinfection dose planning adds complexity, demanding every surface point to receive certain irradiation.

Compared to standard coverage tasks, UV disinfection requires additional dose application, taking minutes to execute [17]. Hence, optimizing disinfection time in known environments is crucial over adapting to unknown terrains or online remapping [15]. Coverage path planning (CPP) aims to calculate a path covering all points of interest (POIs) to envelop the entire Region of Interest (ROI) [19]. CPP efficiency and performance are assessed based on coverage, path distance, completion time, energy use, and obstacle avoidance capabilities [20,21]. CPP ties to various autonomous robotics applications like floor cleaning, mowing, agricultural harvesting, demining, and structural inspections [22].

Ultraviolet disinfection planning can be conceptualized as the modeling and control of light transmission. This process overlaps with similar initiatives in the fields of radiation dose planning, irradiance distribution modeling, and optimization of light placement. Radiation dosage planning outlines methods for measuring and calculating irradiance [23,24,25,26]. Several studies model irradiance distribution, and the rendering of radiosity using the boundary element method describes a method for projecting light rays onto spatial surfaces, quantifying the distribution of energy in space [27,28,29,30]. The optimization of light placements investigates how to deploy the distribution pattern of irradiance sources to ensure irradiance intensity, optimizing the lighting distribution and placement of illuminance sensors within indoor spaces [31,32].

Dose coverage planning entails autonomous robots controlling dosage strategies and planning paths and speeds to uniformly deposit along covered surfaces, similar to radiation exposure planning, Wireless Sensor Network (WSN) layout optimization, and painting robots. The goal is to ensure target dosages while reducing redundancy, waste, and risks. For painting, the entire surface must be coated evenly, with any deviations easily detected visually [33]. Robots achieve uniform painting thickness through spray gun modeling. In WSN layouts, sensor node deployment and network range are crucial for signal coverage, with network topology models balancing node energy consumption and minimizing sensor redundancy [34]. In the optimization of Wireless Sensor Network (WSN) sensor layouts, placing multiple controllers in a large-scale WSN is identified as an NP-hard problem. By controlling the placement and irradiance of lights, the intensity distribution of emitted energy is optimized. The objectives of lighting planning are analogous to our project. Research on lighting optimization [32] and methods for optimizing detector layouts also consider mobile light sources, which, however, are fixed after the design phase. Thus, by strategic planning, sufficient dosage is ensured to the target volume. Efficient photodetector layout methods address the challenges of computational demand and high energy consumption in detector placement.

Previous studies missed two points: the uneven dose distributions creating light redundancy and light use efficiency’s significant impact on robot energy consumption. Addressing this, this paper explores waypoint intervals and time allocation effects on dose uniformity and UVC light energy efficiency. A dose coverage planner is created to guide waypoint placement and disinfection timing.

This paper’s structure begins with key issues in disinfection tasks, drawing insights from other coverage tasks. It constructs a UVC irradiation dose map to inform dose planner modeling, then solves for stationary disinfection waypoints using the MOPSO algorithm. K-means clusters waypoints based on spatial features, with TSP algorithms determining waypoint sequences within subregions for trajectories. Disinfection experiments in various environment sizes compare dose distribution under different irradiation strategies. The results prove that optimized trajectories achieve complete disinfection, enhancing UVC dose coverage efficiency and reducing global dose redundancy. This method offers better performance in energy cost savings, optimizing robots’ light source energy consumption.

## 3. Dose Coverage Planner for UVC Irradiation

This section introduces a novel model for the distribution of deep ultraviolet (UVC) doses, termed the UVC Irradiation Dose Map. Utilizing a 2D grid map, this model computes the cumulative UVC dose across a global map, aimed at assessing dose distribution on environmental surfaces. Considering that the accumulation of UVC dose on object surfaces is feasible, the sequence of execution between waypoints does not affect the disinfection effectiveness. Therefore, when searching for the optimal deployment positions, the focus is solely on the placement of waypoints and irradiation durations, with subsequent separate planning of the paths between waypoints.

### 3.1. UVC Irradiation Dose Map

The dose of UVC provided by the lamps to a point on a two-dimensional surface depends on the radiative intensity of the lamp array $I_{e}$, the distance between the lamp and the surface, and the time of exposure. Let Ω denote the environmental boundaries targeted for disinfection by representing the free space where the robot, with configuration space ∂A, can assume any collision-free configuration. Every infinitesimal surface element ds receives a certain amount of radiant flux every second. The radiation flux distribution model enables the infinitesimal surface ds to receive the following irradiance from lamps with power. The irradiance $D_{k}$ is the total irradiation incident at a surface with area ∂A, and it is computed in terms of the radiant intensity in Equation (2), in which $\left|\vec{r}\right|$ is the distance between the light source and the surface, and cosα is the optical efficiency impacted by the angle between the vector and the surface’s normal $\vec{n}$.

(1) $$E_{e}=\frac{I_{e}\cos\alpha }{|\vec{r}{|}^{2}}$$

The dose $D_{k}$ delivered over a time period $\Delta t=t_{i}-t_{i-1}$ is computed as Equation (2).

(2) $$D_{k}=\int_{t_{k-1}}^{t_{k}}E_{e}dt$$

In the case of focusing on static irradiation at waypoints, assuming a static environment where the relative positions between the lamp and the surface remain constant over time, the energy $E_{e}$ remains constant during time Δt. Substituting Equation (1) into Equation (2), Equation (3) can be expressed, providing the calculation method for each waypoint. The value of $I_{e}$ is obtained from the optical-electric characteristics test of the combined light field in Section 5.

(3) $$D_{k}=E_{e}\Delta t=\frac{I_{e}\cos\alpha }{|\vec{r}{|}^{2}}\Delta t$$

**Figure 2:**
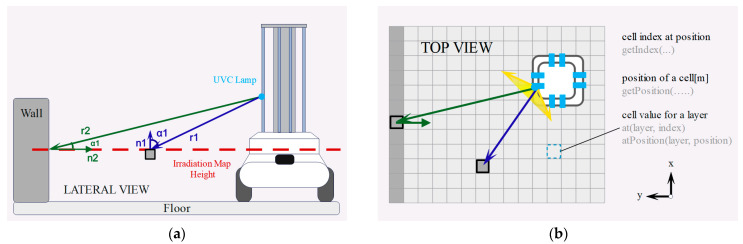
(**a**) Schematic diagram illustrating the calculation method for robot-induced irradiation on object surfaces. (**b**) UVC irradiation dose map constructed based on 2D grid map.

To describe the distribution of dosage on the object surface, we constructed a UVC irradiation dose map based on a SLAM-generated 2D grid map, as shown in Figure 2. We created an irradiation map layer, querying the cell’s index through getIndex(), querying the cell’s position through getPosition(), and querying the cell value through at(layer, index) and atPosition(layer, position). Maintaining a matrix recording the irradiation dosage, after the robot completes static irradiation and disinfection at static waypoints, the dosage obtained by each cell is calculated using Equation (3) and updated into the irradiation map layer. The 2D grid map subdivides space into tiny units, which we use as the smallest units receiving lamp irradiation, with the central position calculating the UVC irradiation dosage representing the entire unit. Because the distance between the center and the edge of the tiny unit is minimal, the approximation error is less than 0.01 µm/cm^2^, which is a reasonable approximation.

Subsequently, as the robot moves autonomously, the accumulated UVC irradiation dose map is updated. With the robot’s position changing over time during patrols, we base our calculations on the 2D grid map obtained from laser scans, which divides space into tiny cells, treated as the smallest units receiving lamp irradiation. The received UVC dose for these small areas is computed, and by updating the robot’s position and calculating the time elapsed since the last update, we traverse each cell of the UVC irradiation dose map, determining the light’s radiative strength and exposure time and incorporating the dose given to each cell as per Equation (3). This accumulated dose is added to the existing irradiation map. Note that only when the cell lies directly within the light field, it is included in the computation of dose accumulation.

Because processing a UVC irradiation dose map represented by a 3D environment is computationally costly and impacts the real-time navigation decisions of the robot, our irradiation map is calculated at a specific height. It utilizes a 2D grid map to identify regions to receive dosage and considers all obstacles merely as walls for simplicity. However, this simplification results in the loss of information about more complex structures, such as multiple surfaces located at different heights but the same x,y position. To address this issue, a boundary strategy was designed to distribute the maximum UVC dose along obstacle boundaries, spreading it across the contour of the entire traversable area to mitigate this shortcoming. The height at which the map is computed and the size of observational cells are configurable. For the most conservative estimate, we can calculate the irradiation map for the ground, as when cells at floor level reach the required dosage, higher ones (between the lamp and the floor) are guaranteed to receive more irradiance, ensuring disinfection throughout the space.

### 3.2. Multi-Objective Optimization Model for UVC Irradiation Planning

#### 3.2.1. Exploration of the Impact of Waypoint Distribution on Dose Coverage Efficiency

The challenge of excessive redundancy during full dose coverage prompted this study to investigate the impact of waypoint spacing on the efficiency of dose distribution. Given that the application of UVC dose requires accumulation over an extended period to reach the threshold, the devised navigation scheme arranges several waypoints for the robot, each featuring a static dwell for a certain duration. Compared to navigation strategies in other works where the robot continuously moves, this approach saves on the energy consumption of the robot’s repeated patrols. The choice of waypoint spacing is critical in arranging static waypoints, as it directly affects the time to achieve coverage and the uniformity of the dose distributed.

**Figure 3:**
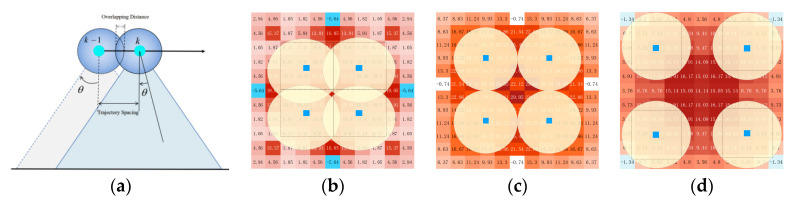
(**a**) The side view of two adjacent waypoints, with waypoints set at different intervals and the robot statically irradiating at each waypoint. (**b**–**d**) LED static irradiation deployed at four waypoints, with varying distances between light sources, exploring the superimposed characteristics of irradiation at the waypoints. The UVC irradiation dose map is presented as a heatmap, and the doses obtained in each observation interval are also recorded.

In this experiment, the overlapping characteristics of irradiation sources were tested to explore how the uniformity of energy absorption depends on the deployment pattern. To better understand the operational effect, four robots equipped with light sources were deployed in the environment to simulate the additive effect after working at four waypoints. All UVC lamps were turned on, and a row of UVC dosimeters was placed in front of the robots. The UVC irradiation dose map was updated over time. The distance between irradiation sources was varied, with lamp positions as shown by the blue dots in Figure 3, set at intervals of 30 cm, 45 cm, and 60 cm. The lamps were turned off once the irradiation dose map showed that cells at the boundary reached the required dose of 16.9 mJ/cm^2^. The times to achieve full dose coverage for the three spacings were 57.4 s, 49.2 s, and 28.9 s, respectively.

Post-closure heatmap results in Figure 3 illustrate the dose distribution obtained in the UVC irradiation dose map after all cells met the dose criteria. The overlay characteristics and evaluation results from the irradiation map were integrated into the next section’s dose planner as constraints for the waypoint strategy, setting the interval for waypoint spacing and the execution time for disinfection.

#### 3.2.2. Evaluation Factor for Dose Coverage Effectiveness

The UVC dose coverage ratio (μ) is introduced to assess the overall completion of disinfection tasks within a spatial environment, based on the statistical analysis of UVC irradiation dose map post-disinfection. The irradiation planner will use these evaluation factors to guide the robotic movement strategy.

The coverage rate indicates the proportion of the UVC irradiation dose map that achieved disinfection, as expressed by Equation (3). Our primary objective in deploying the dose coverage planner is to minimize the risk of contact with COVID-19. We focused on existing studies on the sensitivity of SARS-CoV-2 to UVC, adopting a conservative inactivation dose threshold of 16.9 mJ/cm^2^, denoted as threshold dosage, which results in a 2-log reduction (99% inactivation) of the virus.

(4) $$\mu =\frac{\sum_{cell}\delta_{ij}}{num}$$

(5) $$\delta_{ij}=\{\begin{array}{ll} 1, & \left(D_{k}-D_{threshold}\right)\geq 0\\ 0, & otherwise \end{array}$$

If a cell reaches the dose threshold, it is considered covered. The number of cells is determined by the resolution setting of the grid map. Only when each cell in the entire environmental irradiation dose map has received a dose above the threshold will the coverage rate reach 100%. A μ greater than 100% indicates that the actual surface area of the objects receiving the dose exceeds the real area; i.e., the coverage meets the standard. During dose planner execution, achieving the coverage rate is a constraint to ensure the successful completion of the entire scene’s dose coverage task.

To quantify wasted light energy, we define global dose redundancy on the UVC irradiation dose map, denoted as dose redundancy, as shown in Equation (6). It calculates the difference between the actual dose each cell receives and the required threshold dosage, reflecting the extent to which the actual dose exceeds the amount needed to inactivate the virus. During the operation of the dose coverage planner, we aim for lower global dose redundancy to reduce unnecessary energy consumption by the UVC lamp arrays.

(6) $$D_{r}=\sum_{k=1}^{num}D_{k}-D_{threshold}$$

We found that the uniformity of the UVC irradiation dose map is strong evidence of energy waste in existing coverage solutions. Specifically, in disinfection operations within a static light field, the dose on environmental edges is often met at the expense of efficiency, leading to several times more energy in areas close to the static light source. Previous research often relied on manual repositioning of fixed light sources by operators to increase the uniformity of dose distribution. The mean squared error reflects the uniformity between global grid doses and is calculated as the sum of squares of the deviations from the threshold dose across all global cells, averaged according to Equation (7).

(7) $$D_{MSE}=\frac{1}{num}\sum_{i=1}^{num}{\left(D_{k}^{i}-D_{threshold}\right)}^{2}$$

Dose coverage efficiency (Equation (8)) measures the portion of the working lamp energy that effectively contributes to reaching the threshold dosage.

(8) $$\eta_{DCE}=\frac{\sum_{cell}D_{k}}{\sum_{cell}\delta_{ij}*D_{threshold}}$$

A high dose coverage efficiency indicates a higher proportion of the emitted energy serves its practical purpose rather than being superfluous. Greater efficiency in UVC dose coverage leads to minimized dose redundancy and optimized energy consumption.

#### 3.2.3. Multi-Objective Optimization Model for UVC DCP

Building on the previous section’s insights, we addressed the critical steps for optimizing energy consumption in a mobile robot’s UVC dosage coverage task. Key goals include reducing redundancy in the UVC irradiation dose map and enhancing the uniformity of dosage distribution from a local observation perspective, all the while ensuring adequate surface coverage. The objective function of the dose coverage planner proposed herein is designed to optimize these aspects. However, it is acknowledged that these optimization targets may lead to divergent paths for the planner. Our aim is to find a solution guided by the synergy of these factors.

(9) $$\begin{array}{l} \begin{array}{cc} \;argmin & \;\omega_{1}\eta_{DCE}+\omega_{2}D_{r}+\omega_{3}D_{MSE} \end{array}\\ \begin{array}{ccc} & \;s.t. & \mu \geq 100\% \end{array}\\ \begin{array}{ccc} & & dis(\Delta x_{k},\Delta y_{k})\leq gap_{\max},(x_{k},y_{k})\subseteq \Omega \end{array}\\ \begin{array}{ccc} & & num\leq S_{Area}/gap_{static} \end{array}\\ \begin{array}{ccc} & & \sum_{cell}t_{k}\leq t_{static} \end{array} \end{array}$$

MOPSO is extensively utilized for solving multi-objective optimization problems by identifying and updating the global optimal solution set through the discovery of Pareto-dominant solutions. In the MOPSO dosage coverage planning, a conflict exists between light efficiency and the uniformity of dose distribution, while the trends of dose redundancy and uniformity are consistent. Consequently, light efficiency is designated as the objective function $f_{1}$, and a combination of dose redundancy and uniformity forms the objective function $f_{2}$. A penalty factor is designed to balance their impacts and manage the rhythm between local and global coordination. Light efficiency is determined based on the effects of waypoint spacing on irradiance characteristics, informed by empirical experience. Additionally, constraints are imposed on the static irradiation duration at each disinfection waypoint to prevent exceeding the irradiation time of a point light source in single-point mode; constraints are also set on the spacing between waypoints based on accumulative properties analyzed through empirical regularities. Furthermore, considering the irradiation characteristics of UVC light, both single-point and multi-point overlay modes exhibit a defined effective irradiation range. The particle movement step length in each iteration is constrained by the distance between two points, ensuring it is less than the minimal efficiency gap during the overlay irradiation process. The number of disinfection waypoints is a fixed value calculated based on the map size and grid resolution, with larger maps requiring more waypoints. Achieving complete dosage coverage and satisfying coverage rate remains the primary constraint.

### 3.3. Implementation of the MOPSO Algorithm for Solving the UVC DCP

#### 3.3.1. Solution Procedure and Parameter Configuration

The number of waypoints is determined by the environmental map size and the effective distance gap of single-source static irradiation. The sum of disinfection times at each waypoint must not exceed the standard duration $t_{k}$ for single-source irradiation. These constitute the fitness function of the MOPSO algorithm.

Secondly, compute the MOPSO fitness function as Equation (9). The parameter settings for the MOPSO algorithm are in Table 1. Within the range of waypoint numbers, calculate the dosage applied by all waypoints to the current cell and update the UVC irradiation dose map according to Equation (3). Subsequently, initiate the global calibration phase, moving particles and recalculating the cost. As favorable configurations of waypoints are updated, the UVC irradiation dose map and cost functions are similarly revised. Non-inferior solutions are preserved in an external archive (i.e., Archive set) based on the Pareto dominance relationship to ensure all non-inferior solutions are retained. Compute non-inferior solutions based on their distance in objective space and select a global guide for the particles. Update the particles’ individual historical best, individual guides, and the Archive set based on the fitness function results. The process concludes when the position difference of waypoints converges, and the cost function plateaus. After convergence, the algorithm returns the solution from the external archive as the final waypoint strategy. The flowchart of the MOPSO algorithm is as shown in Algorithm 1.
**Algorithm 1.** MOPSO solves the coverage planner**Input:** Environmental grid map Ω **Output**$:\;\mathrm{Waypoints}\;\mathrm{location}\;P=\left\{x_{1},\ldots ,x_{n}\right\}$, static disinfection time $T=\left\{t_{1},\ldots ,t_{n}\right\}$
1:Get set of unoccupied cells ${U=\{u}_{1}{,\;u}_{2},\ldots {,u}_{n}\}\subset \Omega$, Initialize Position of Waypoints $P_{0}\subset U$$,\;\mathrm{static}\;\mathrm{disinfection}\;\mathrm{time}\;T_{0}$
2:**For** Cell i = 2 to I, traverse waypoints P to irradiate $U_{i}$
3:  **while **$p_{k}$ in P do4:     Set the optimal Static waypoint disinfection time $t_{k}$
5:     **Compute **$\;\mathrm{the}\;\mathrm{dose}\;D_{k}$ exerted by waypoint $p_{k}$$\;\mathrm{on}\;U_{i}$
6:   **End while**
7:  **Update **$U_{i}$ to global dose irradiation map8:  **Compute** fitness for all observation cells9:**End For**10:**Repeat** 2–9 within the range of particle quantity. Calculate the personal best position for each particle11:Determine the global best position for the entire particle swarm12:**Update** the positions and velocities of the particles based on guidance13:Filter out non-dominated solutions based on Pareto dominance relation and add them to the Archive set14:Waypoints Collision detection15:**Repeat** 10–14 until convergence16:**Return** Waypoints location P, static irradiation time T in external Archive set

#### 3.3.2. Analysis of Convergence Properties

For the comparative analysis, we selected classic metaheuristic algorithms renowned for handling multi-objective optimization problems, namely MOEA/D, NSGA-II, SPEA2, and PAES, to conduct convergence analysis of objective functions. To mitigate the impact of heuristic algorithm randomness on results, the outcomes of each algorithm were averaged over 20 experiments. To ensure fair comparison, we utilized parameter values recommended in the literature for all compared algorithms [35,36].

**Figure 4:**
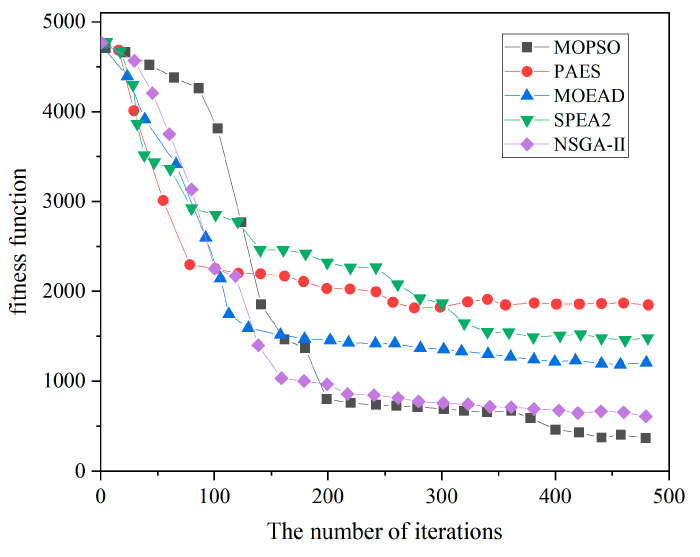
Convergence comparison of five metaheuristic multi-objective optimization algorithms.

The convergence speed and convergence effectiveness of optimization algorithms are important metrics for evaluating algorithm performance. Figure 4 presents the convergence analysis curves for the fitness function of five optimization algorithms in solving the dosage coverage planning model. Among them, PAES demonstrates the fastest convergence speed, followed by MOEDA, while MOPSO and NSGA-II have similar speeds. SPEA2 initially shows excellent performance but slightly lags in the later stages. In terms of convergence effectiveness, although PAES converges quickly, it does not achieve the best value for the fitness function. MOPSO and NSGA-II are comparable in their convergence outcomes. Overall, while the convergence speeds are closely matched among the algorithms, MOPSO slightly excels in the final convergence effectiveness of the fitness function, yielding the most favorable results.

It is worth noting that our focus extends beyond merely observing the convergence rate of metaheuristic algorithms in solving multi-objective models. We delve into analyzing their suitability in handling multiple conflicting objectives and achieving a balanced synthesis of optimal solutions. Alongside discussing convergence, we explored the applicability of the five metaheuristic algorithms in solving irradiation planning multi-objective optimization models by plotting their Pareto frontiers.

**Figure 5:**
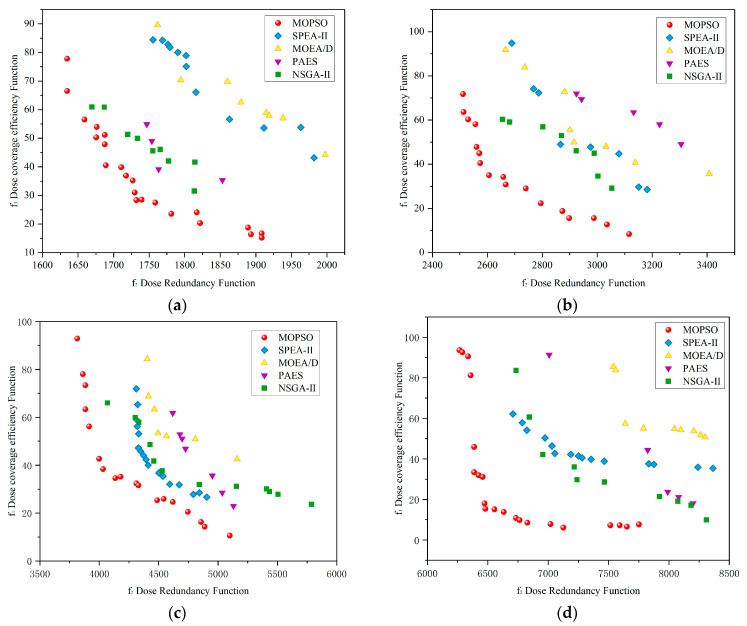
Metaheuristic algorithms addressing map tasks of varying areas to solve the Pareto optimal front of the irradiation multi-objective model. (**a**) Map area 15 m^2^. (**b**) Map area 20 m^2^. (**c**) Map area 25 m^2^. (**d**) Map area 30 m^2^.

Figure 5 illustrates the Pareto frontiers of the non-dominated solution sets obtained by the five algorithms for four objectives. There are differences in the search adaptability exhibited by the algorithms when solving objectives on maps of different sizes. Through multiple experiments, compared to the four contrastive algorithms, MOPSO generated a more evenly distributed and closer-to-origin non-dominated solution set, indicating its stable performance in solving dose planning models. In Figure 5d, although MOEA/D yielded smaller irradiation redundancy (f1), it came at the cost of uneven dose distribution, reflected in its poor performance in objective function 2. Additionally, SPEA2 exhibited lower f2 irradiation redundancy in large-scale map objectives, slightly outperforming MOPSO’s results. For small-scale map objectives (e.g., Figure 5a), PAES and NSGA-II demonstrated fairly good search capabilities, but PAES performed poorly for large-scale map objectives (e.g., Figure 5d). Among all algorithms, MOPSO obtained more non-dominated solutions for minimizing irradiation redundancy objectives across various objectives.

## 4. Disinfection Path Planning Based on Spatial Segmentation

In the preceding section, we solved the coverage planner using the MOPSO (Multi-Objective Particle Swarm Optimization) algorithm, which provided the waypoint coordinates and the static dwell disinfection times at each point. For a simple polygonal map without obstacles, a spiral path can be employed. However, when dealing with irregularly shaped or unstructured terrains, obstacle avoidance and regional segmentation are necessary. In large-scale settings where the map regions have distinct features, and the number of waypoints is substantial, it is essential to execute the path in segmented areas.

**Figure 6:**
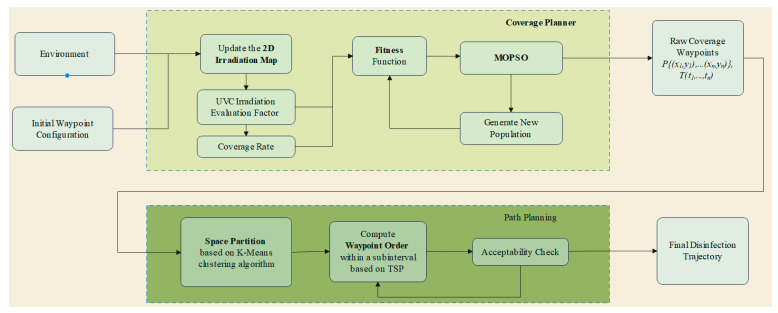
The hierarchical framework of overall disinfection task planning, illustrating the execution flow of the irradiance planner and path planning algorithm. Given the initial population parameters for the robot, environment data, and three types of motion strategies, the best value is searched by simulating each individual within the population. MOPSO is executed to obtain waypoints and static dwell disinfection times. Sub-regions are divided based on the K-means algorithm, and finally, the TSP algorithm is used to determine the sequence of waypoints, generating an optimized trajectory.

In this section, we incorporate consideration of the environmental map’s structural attributes to generate the final navigation path. Firstly, the waypoints are clustered based on the structure of the map; then, aiming to minimize the overall movement cost, the optimal sequence of waypoint assignments is calculated, resulting in the disinfection trajectory. The hierarchical framework of overall disinfection task planning is depicted in Figure 6, illustrating the execution flow of the irradiance planner and path planning algorithm.

### 4.1. Spatial Segmentation Based on K-Means Clustering Algorithm

**Figure 7:**
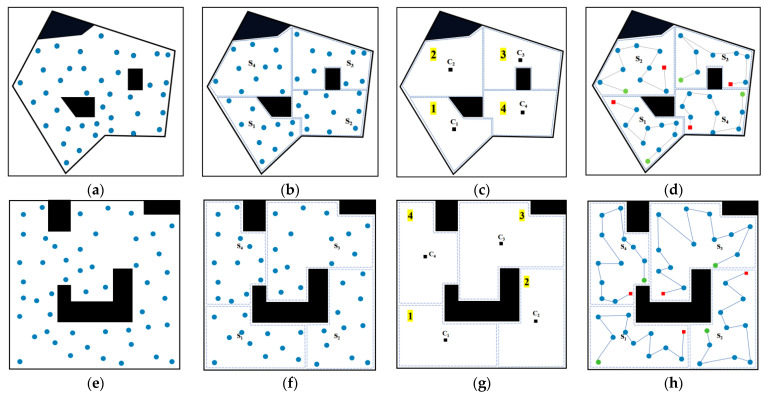
Simulation of DCP planner in two structured obstacle terrains. (**a**,**e**) Waypoint locations after the dose coverage planner is executed by MOPSO (blue circle). (**b**,**f**) K-means clustering of waypoints, with division into cluster groups S_1_ to S_4_. (**c**,**g**) The clusters are ordered based on the robot’s starting location and cluster centroids, demonstrated in the highlighted yellow box. (**d**,**h**) Performing TSP on the sequence of waypoints within each group to obtain the ordered flight trajectories. Additionally, determining the starting point (green circle) and ending point (red square) of each subcluster based on the order of the groups.

We utilize the K-means clustering algorithm, an unsupervised machine learning technique, to segment waypoints. The selection of K-means is based on its capability to fix the number of required clusters, K, and group nearby sample points according to their Euclidean distances. Initially, the location of the segment centroids is estimated, one at a time, until the number of segments equals the fixed value. Group centroids, cj, are randomly assigned and each sample point’s distance to the centroids is compared. Each sample point is then assigned to the closest centroid group. The group centroids, cj, are recalculated by taking the average of the sample points, pi, within the corresponding group until the centroids converge, minimizing the sum of squares within each group. This process ensures that a cluster of K sample points, {S_1_, S_2_, …, S_k_} ⊆ S, is obtained, as shown in Figure 7b.

Additionally, the sequence between groups is determined to facilitate smooth transitions between clusters. Considering the robot’s starting position and the centroids of each cluster, the cluster sequence is computed using the Depth-First Search (DFS) algorithm. As displayed in Figure 7c, the robot first visits group S1, followed by S_2_, S_3_, and S_4_.

### 4.2. Concatenation of Disinfection Pathway Waypoints Utilizing the TSP Algorithm

Once the spatial segmentation of groups is attained, the individual waypoints are subdivided into cluster sets, P. To create a continuous path connecting all clusters, the last sampling point of each cluster is set as the starting point for the subsequent cluster until completion of the last one. Within each cluster, the Traveling Salesperson Problem (TSP) is executed to determine the waypoint sequence for the sub-region. Following the order of clusters, a continuous sequence of waypoints from the robot’s initial position is established to execute the irradiation path.

The TSP algorithm is commonly used to address the sequence problem of waypoints in path planning contexts. We implement the TSP algorithm through a greedy approach. The starting point for each round is chosen from the waypoint set closest to the robot’s initial position or the end-point of the previous collection. Inputting the coordinates of the waypoints P, every two waypoints are distance-calculated and a distance matrix is constructed. The backtracking method is used to generate all possible waypoint permutations as the solution space. Subsequently, the distance between each adjacent waypoint is located using the distance matrix, summing the total length of the waypoint path. The shortest path is selected as the optimal solution based on path length. Upon determining the sequence of waypoints, the optimal path is outputted. Figure 7d illustrates the sequential execution of irradiation mapping from the robot’s initial location.

## 5. Disinfection Experiment

To assess the disinfection efficacy of the coverage planner, we conducted a disinfection experiment within a laboratory interior of approximately 60 m^2^. Using a UVC radiometer (Linshang UV Light Meter LS126C, Shenzhen Linshang Technology Co., Ltd., Shenzhen, China), we measured the radiation intensity applied by the robot and evaluated the disinfection effect by examining the dose distribution in UVC irradiation dose map. We designed assessment metrics for the disinfection trajectory to answer the following questions:

- How much better in terms of energy savings (coverage rate, dose redundancy) is the optimal planning disinfection trajectory compared to other traditional strategies?
- How universal is it against various viruses? Are there differences in the effects of executing trajectory planners?

### 5.1. UVC LED Lamp Array Irradiation Photoelectric Performance Test

The disinfection robot is equipped with AlGaN-based UVC LED chips, chosen for their high radiation efficiency and strong photoelectric conversion performance, aiming to achieve optimal ultraviolet irradiation capabilities. The lamp array consists of eight UVC LED strips arranged in a surround-style distribution structure, with heat sinks and exhaust fans installed in the middle to alleviate the heat generated by prolonged irradiation of the high-power LED lamp array. Initially, we tested the irradiation capabilities of both individual lamps and the overall lamp array, and based on these results, computed and updated the accumulated UVC irradiation dose map as the robot moves.

**Figure 8:**
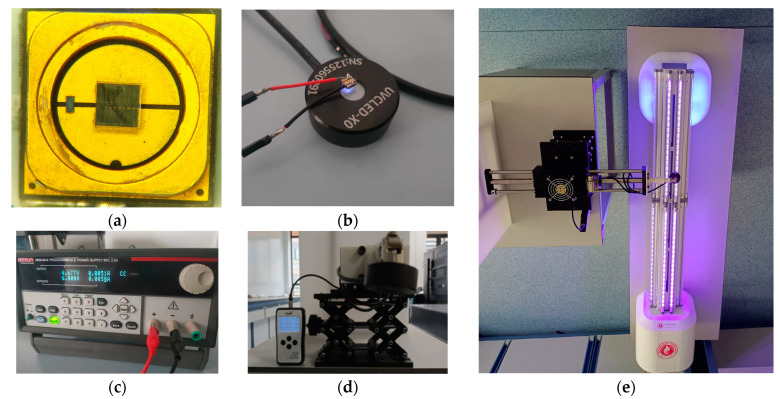
Irradiation performance experiment. (**a**) AIGaN-based UVC LED chip, (**b**) UVC measurement probe, (**c**) constant current power supply, (**d**) irradiance measurement instrument, (**e**) light assembly with mounted AIGaN-based UVC LED chips.

The radiant performance of the UVC LED single lamp (Shenzhen Deshengxing Electronics Co., Ltd., Shenzhen, China) (shown in Figure 8a) was tested using the constant current source depicted in Figure 8c, with LED input stepped current excitation, resulting in the radiation parameters shown in Table 2. In the subsequent sections addressing the design and application of the overall lamp array, the UVC LED operates at a rated current of 500 mA, as illustrated in Figure 8e.

Under a 500 mA excitation, the peak wavelength λp measures 273 nm; the radiant flux $\phi_{e}$ is 77.432 mW, with a deep ultraviolet irradiation photon-to-electron conversion efficiency of 3.3175%. Each UVC LED strip has a rated power of 72 W, with the overall rated electrical power of the entire lamp array being 576 W. With a photonic chip luminous efficiency of 3.3%, the effective UV output of each strip is 2.38 W—this figure pertains specifically to the UV component effective in pathogen inactivation, summing to an overall effective optical power of 19 W for the system.

**Figure 9:**
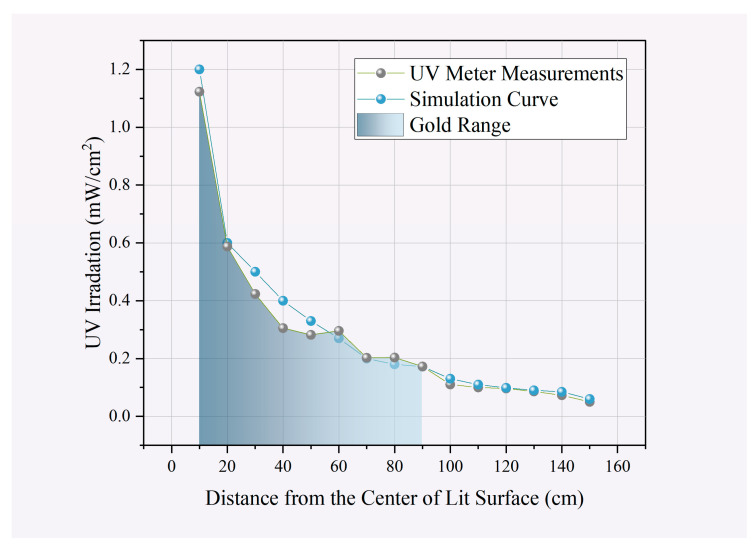
Static irradiation characteristics curve of combined UVC light field. Experimental measurements of irradiation power from UVC light sets at different distances.

Nonetheless, to approximate real-world scenarios more accurately, where robots equipped with a combined light field move, static irradiance measurements were conducted. Irradiance strength was measured at different distances vertically, as shown in Figure 9. The article uses actual measured data to revise the theoretical formula of irradiance power (mW/cm^2^). The experimentation results include the following graphical representations: Experimental Data, Best-Fit Curve, and Theoretical Curve. The green-shaded area represents the effective irradiation range, while beyond it, the intensity falls short of the level required to deactivate viruses. Hence, to make more effective calculations, when adjusting the waypoints under the guidance of the dose planner, referencing it establishes limiting constraints.

### 5.2. Experimental Platform and Testing Methods Introduction

**Figure 10:**
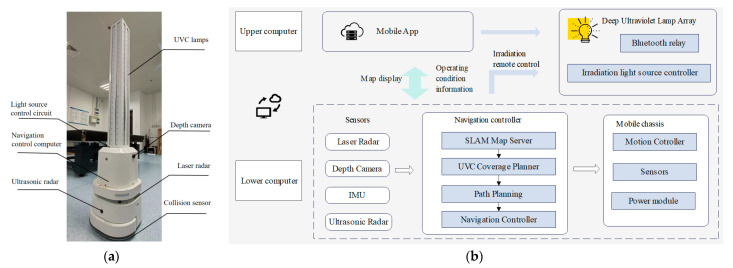
(**a**) The overall structure of the deep ultraviolet disinfection robot. (**b**) Distributed control structure and communication mechanism.

The core component of the robot control system is an NVIDIA Jetson TX2 industrial computer (NVIDIA Co., Ltd., Santa Clara, CA, USA) (featuring a dual-core NVIDIA Denver™ 64-bit processor and quad-core Arm^®^ Cortex^®^-A57 MPCore processor), capable of meeting real-time navigation requirements. The robot is equipped with various sensors including a 2D LiDAR (SLAMTEC RPLIDAR S3, Shanghai Slamtec Co., Ltd., Shanghai, China), depth camera (Orbbec 335, Shenzhen Orbbec Technology Co., Ltd., Shenzhen, China), IMU (MPU6050, InvenSense, Inc., San Jose, CA, USA), and ultrasonic sensors (HC-SR04, Guangzhou Xindejia Co., Ltd., Guangzhou, China) to gather environmental information. The main controller processes sensor data to execute mapping, planning, and navigation tasks, and controls the chassis motor drive board via CAN communication. The Apollo robot chassis developed by SLAMTEC (Shanghai Slamtec Co., Ltd., Shanghai, China) is utilized, with motion chassis motor drive achieved through an STM32 controller (stm32f103c8t6STM, STM Co., Ltd., Geneva, Switzerland). The task scheduling relationships within the disinfection robot control system are illustrated in Figure 10. Energy data were acquired through a UVC ultraviolet detection instrument (Linshang UV Light Meter LS126C with a UVC-X0 probe) used for real-time intensity and energy testing of the light source, with a spectral response range of 260 nm–285 nm.

Autonomous intelligent control is implemented based on the ROS (Robot Operating System) robotics framework. A dedicated ROS package for executing the coverage planner has been developed and navigation systems deployed. Within the ROS operating environment, RTAB-Map is configured for simultaneous localization and mapping (SLAM), facilitating the construction of 2D grid map and obtaining the robot’s position in the global map [37]. The mapping thread receives sensor data from the LiDAR and depth camera, generating the 2D grid map required for irradiance mapping. Subsequently, the main controller executes the dosage coverage planner (DCP), obtaining static disinfection waypoint coordinates for subsequent navigation thread trajectory calculations.

Following this, the move_base node receives the coordinates of adjacent disinfection waypoints from the coverage planner, defining these as the endpoints for the planned global route. It then employs the A* algorithm to meticulously chart the trajectory between these waypoints. Subsequently, the path is refined using the DWA local planning algorithm to enhance its feasibility and accuracy. Ultimately, the move_base node translates this refined path into precise chassis control commands, which are converted into motor control signals, culminating in the output of PWM signals to execute the desired motion efficiently. Additionally, an upper computer APP software (GXU Disinfection Robot Navigation Control software, version 3.2.1) has been designed, enabling staff to remotely control robot movement and toggle UVC lamp groups. For safety during disinfection operations, the robot’s mounted camera and ultrasonic module continuously perform target detection. Upon detecting pedestrian movement targets, the robot halts movement and deactivates the UVC irradiation lamp group.

### 5.3. Autonomous Mobile Disinfection Experiment

**Figure 11:**
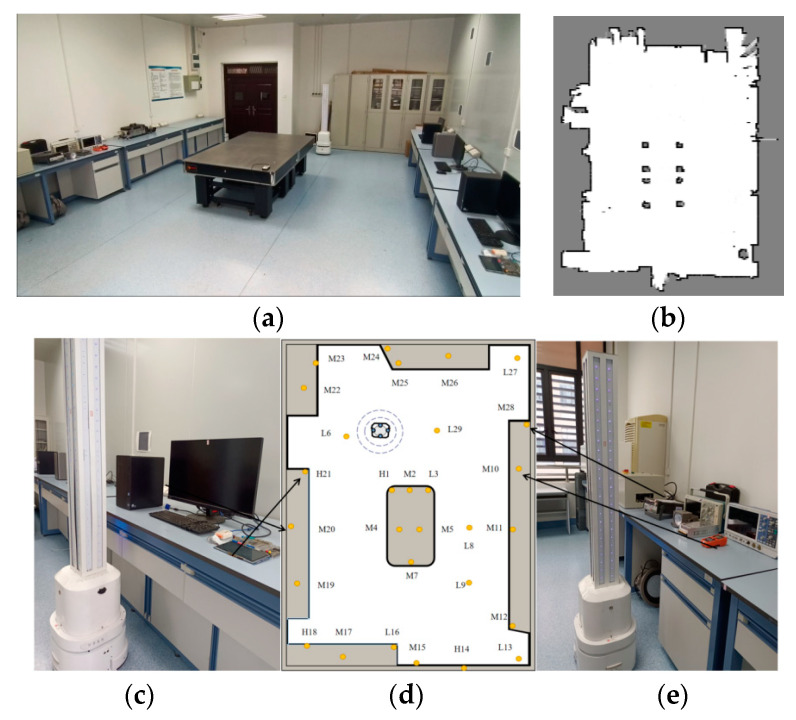
Autonomous disinfection experiment for mobile robots. (**a**) Laboratory scenario. (**b**) 2D grid map acquired after performing slam. (**c**,**e**) The marker location where the illuminometer is placed. (**d**) Floor plan of laboratory scene simulation, where orange dots represent illuminometer test points.

**Figure 12:**
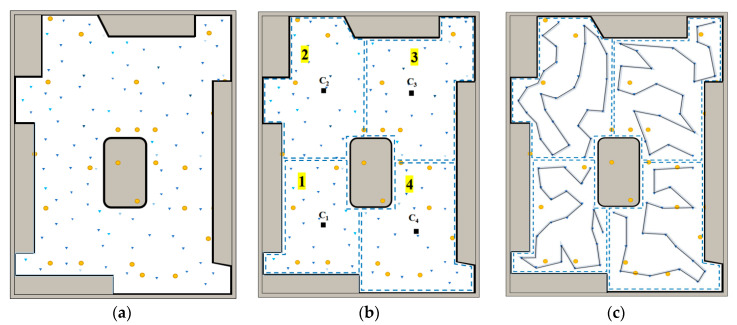
Disinfection in the laboratory and execution process of the planner: (**a**) The planner generates waypoints and corresponding timestamps. Triangles indicate the locations of the waypoints, and the shading represents the duration of disinfection at each waypoint. (**b**) K-means clustering divides the waypoints into regions. In addition, the areas marked in yellow indicate the execution order of movements between clusters, where c1 to c4 represent the centroids of each cluster. (**c**) The TSP algorithm is executed to generate the path.

We executed the DCP (dose coverage planner) in the laboratory environment. The finer the resolution of the grid map, the more accurate the computed final dose will be, leading to better planner performance. However, the cost of updating the UVC irradiation dose map increases linearly with the level of division accuracy. Taking into consideration the real-time nature of planner decisions, and after comparing the pre-test effects of different segmentation sizes, we have selected a 15 cm scale for the observation of the UVC irradiation dose map. The ROI is divided into cells of size 0.15 m × 0.15 m. The DCP planner is then applied on the UVC irradiation dose map, and the resulting waypoint strategy is shown in Figure 11a, with triangle markers indicating waypoint positions and color intensity reflecting decontamination time. The next step involves dividing the region and performing path planning to connect the waypoints. A mean clustering with k = 4 is used for region division. The execution order of the regions is determined based on the initial pose of the robot. After obtaining the waypoint sequence using the TSP algorithm for each region, the robot’s final global motion path is generated. The scenario in Figure 11 was used as the test sample for evaluating the effectiveness of three different path planning algorithms for UV irradiation, as depicted in Figure 12.

(1) The spiral fixed spiral method is commonly employed to plan paths on convex polygons as it imposes low computational demands on the robot platform [4]. The spiral planner guides the robot to move in a repetitive pattern within the environment along a fixed trajectory. However, it fails to consider how dose accumulates in the environment and maintains a constant speed of 0.1 m/s during navigation.

(2) The APF(Artificial Potential Field) algorithm is widely used for complete coverage tasks in path planning [17]. It utilizes artificial forces simulating the interaction between objects to guide the planning process. The coordinates of the start and target points are determined, and suitable potential field attributes are assigned to each obstacle. The potential field is calculated by computing the repulsive forces exerted by each obstacle and combining them with the attractive force between the start and target points. The repulsive forces are determined based on the distance between obstacles and path points. The path points are updated by considering the resultant force derived from the current position and the potential field.

(3) Voronoi-based path planning is generally applicable in environments with obstacles, making them the safest areas for robot traversal [16,38]. The Voronoi diagram is constructed using the robot’s current position as the initial point. The robot then follows along the boundaries connecting adjacent Voronoi cells to execute the path. At the centroid of each Voronoi cell, the robot halts to perform static disinfection.

#### 5.3.1. Dose Distribution Analysis

**Figure 13:**
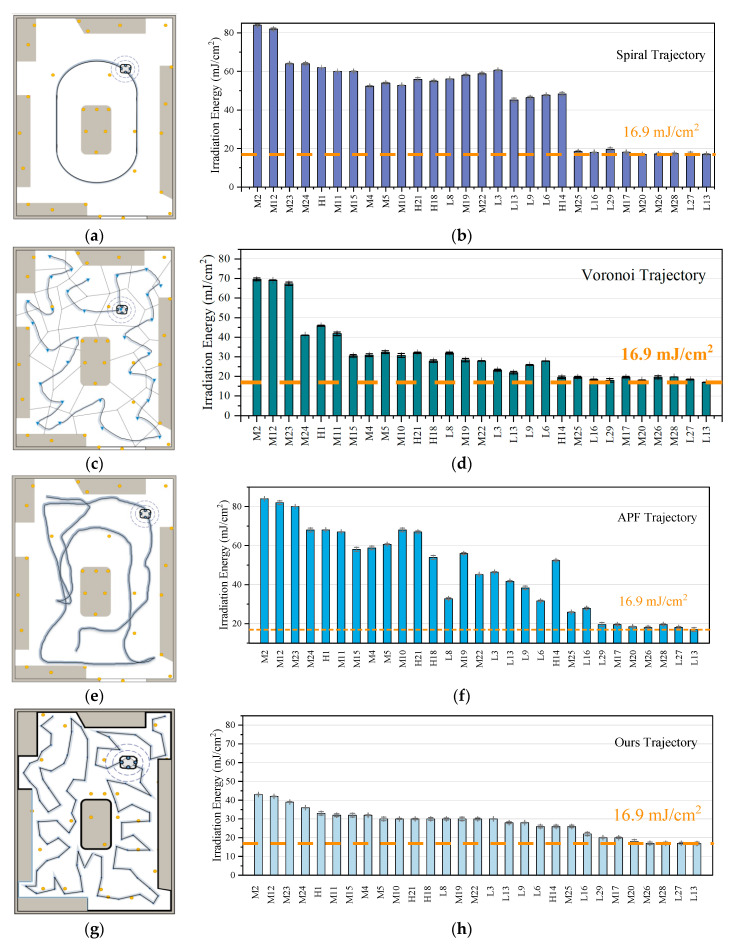
Comparative experiment: The experiment compared the dose coverage effect after executing four path algorithms. (**a**,**b**) Spiral. (**c**,**d**) Voronoi. (**e**,**f**) APF. (**g**,**h**) Ours. The illumination energy measurement device was deployed at the observation points in Figure 8d, and the robot executed the trajectory according to the algorithm in the left figure. After the disinfection, the distribution of UVC dose at each point was measured as shown in the right figure. For easy comparison, the abscissa adopts a unified labeling order, and the ordinate adopts proportional scale.

We conducted experiments with different disinfection algorithms in real scenarios and evaluated the disinfection efficacy, as shown in Figure 13 and Table 3. We calculated the total time for robot movement, static irradiation, and execution of repositioning. Since the robot runs the UVC LED light group throughout, and the luminous energy remains basically unchanged over time, the disinfection time also reflects energy consumption. The work includes the time for the robot to move between waypoints and the time for static irradiation at the waypoints. The total dose redundancy is obtained from the difference between the measured dose at the observation point and the inactivation threshold. The peak-to-peak value and standard deviation of the dose distribution at the observation points reflect the uniformity of the disinfection dose deployment. During the operation of the robot, periodic repositioning is carried out to ensure the accuracy of the navigation trajectory, which results in a deviation between the pose at the waypoint and the simulation, referred to as navigation position error. The deviation between the robot’s pose and the trajectory leads to discrepancies in the dose application and simulation calculation. Each algorithm’s trajectory was repeated five times, each with a fixed initial robot pose. Additionally, before starting navigation, the robot always performs a 360-degree turn to draw a grid around its initial position, minimizing the error caused by the initial pose.

The standard spiral path strategy exhibits low trajectory errors during navigation. This strategy can quickly calculate the trajectory configuration of the entire environment map, with no risk of collision with obstacles. However, simply exploring the environment with a fixed trajectory and speed, without considering the required disinfectant dosage, is clearly not a good strategy. The spiral strategy did not achieve complete dosage coverage due to its fixed trajectory distance from the environment boundary. To increase the dosage, the only option is to increase the working time and the length of the looping path.

The Voronoi-based trajectory planning in a map with obstacles has a similar moving path length to that in this paper. It statically executes irradiation at the centroid of each interval, resulting in the shortest path length for moving. However, due to its uneven segmentation, the dosage coverage has a significant difference in intensity at the unequal length boundaries, leading to overall high redundancy. Additionally, the algorithm deployment based on Delaunay triangulation leads to a long computation time. The APF has the shortest moving time, but the deployment error during navigation leads to substandard coverage; i.e., there are dosage coverage gaps. In fact, when we conducted simulations, the irradiation time calculated for this method could achieve complete dosage coverage. However, due to the need for multiple relocations, the positional offset during navigation introduces deviations between dosage application and simulation calculation.

The deployment of the approach in this paper significantly reduces the redundancy of global dose observation, as the planner reasonably controls the waypoint spacing, weakening the pattern of low-dose irradiation over long distances. Additionally, in irregular edge environments, redundancy is reduced by controlling waypoint time. By controlling time and achieving uniformity in irradiation time and dosage distribution, significant advantages are demonstrated in achieving complete surface disinfection.

#### 5.3.2. Disinfection Energy Consumption Analysis

The energy consumption for disinfection P is divided into two main components: the energy used for movement $P_{motion}$ and the energy used for irradiation $P_{irradiation}$. The entire duration of disinfection is considered for calculating the irradiation energy since the lights remain continuously on, with each UVC LED lamp strip rated at 72 W and the total rated power of the lamp array at 576 W (equivalent to an energy consumption of 2073.6 kJ, converted from watts to joules per second). In analyzing the energy consumed by movement, the standby rated power at waypoints is 32 W, while the rated power during movement is 39 W. Ultimately, the total energy consumption (in kJ) is converted into electrical energy (in kWh).

Evaluation of disinfection energy consumption efficiency in Table 4. Due to differing methodologies, both the trajectory planning based on the Voronoi diagram and the algorithm discussed in this paper utilize a combined approach of static disinfection at waypoints and movement along a trajectory. The time required is composed of the duration of movement and the static irradiation time. Both the spiral and the Artificial Potential Field (APF) strategies employ purely dynamic movement schemes. The advantages of combining static and dynamic movements become more apparent in irradiation tasks that require a certain duration to achieve the required intensity. The energy consumption calculations in this paper are based on the rated power of the robot and are subject to certain errors; however, the trends reflected in the comparative results are quite clear, showing that the use of an irradiation planner in disinfection tasks significantly reduces energy consumption.

### 5.4. Disinfection Targeting Various Pathogens

According to research conducted by the University of Porto [1], the inactivation irradiation thresholds for common pathogens are summarized in Table 5. Since pathogens in real-life scenarios are not found in isolation, we have configured the robot to operate in both high-dose and low-dose modes to address a wide range of disinfection irradiation requirements. The irradiation threshold for the high-dose mode is set at 100 mJ/cm^2^, while the low-dose mode operates at 20 mJ/cm^2^.

**Figure 14:**
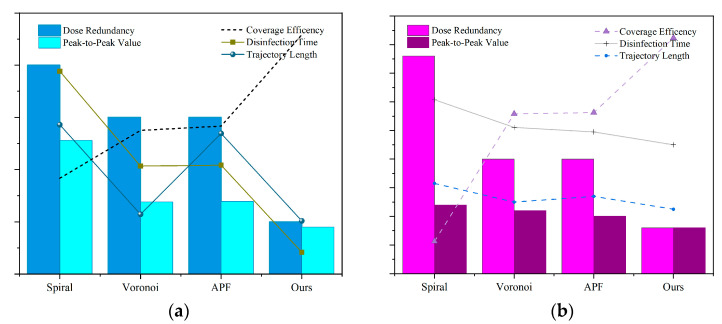
Sensitive analysis of dose coverage planning algorithm to high- and low-dose targets. (**a**) Low-dose mode, 20 mJ/cm^2^. (**b**) High-dose mode, 100 mJ/cm^2^.

Sensitivity analysis of dose coverage planning algorithm for high- and low-dose targets in Figure 14. In the low-dose mode, the dose redundancy and dose peak-to-peak differences among algorithms are relatively small. However, in high-dose threshold disinfection tasks, the planner proposed in this paper demonstrates significant advantages, particularly in terms of dose redundancy and irradiation time effectiveness. This results in outstanding performance in reducing irradiation energy consumption. Such performance is influenced by the characteristics of deep ultraviolet irradiation, where the differentiation increases with dose intensity. In contrast, in other methods, due to the movement of robots along the centroid of the partition for irradiation, there is a significant difference between the irradiation center and the edge, leading to a significant increase in dose peak-to-peak values and dose redundancy. This fully demonstrates the effectiveness of the DCP planner, as its set mean square deviation constraint factor acts as resistance against waypoint redundancy.

## 6. Conclusions

The goal of disinfection planning is to calculate the trajectory of the autonomous mobile robot and the UVC irradiation application strategy in order to cover the entire surface with the required dose. Disinfection planning is a special case of robot motion planning, with the additional complexity that every point on the surface of the object must reach the dose threshold. However, inactivating viruses requires a high intensity dose, which results in significant energy consumption. Specifically, if the energy is exhausted during the task execution and the robot has not completed the disinfection task, not only are the previous efforts wasted, but there is also a risk of human exposure to the virus as the robot needs to enter the scene for recovery and recharging operations. This poses a challenge for large-scale disinfection tasks for the robot. The energy consumption of the light source during the disinfection task is far greater than that of the chassis.

Given the computational constraints of robotic systems and the precision of their mobile execution, the ultraviolet irradiation planning algorithm presented in this paper is exclusively applicable to disinfection tasks on 2D surfaces within static environments. In principle, these tasks are performed in unoccupied indoor settings to prevent UV exposure from harming humans. For safety, this study also introduces an emergency response protocol for sudden environmental changes; the motion halts and the UV lights are deactivated when sensors detect dynamic objects or when proximity to an object falls below the collision threshold. Planning for dynamic environments involves complex nonlinear systems. Future work may incorporate fuzzy control to better adapt to the system’s dynamic changes [39]. For instance, fractional order learning algorithms could more accurately model the nonlinear relationships and converge more swiftly and precisely to the desired state. Furthermore, the algorithm does not consider the characteristics of non-holonomic vehicle models, resulting in somewhat rigid navigation actions. Future work could expand to more complex scenarios by introducing non-holonomic constraints to further optimize robot motion [40,41,42,43,44].

We present a novel dose coverage planner for mobile robot disinfection tasks. This study investigates the accumulation characteristics of UVC irradiation on surfaces and incorporates it into the deployment constraints of waypoints to optimize the use of UVC energy and reduce dose redundancy. To address the issue of energy waste, we establish a UVC irradiation dose map to control the dose applied to the surface by planning the coverage strategy of the combined light field in different positions. We conducted experiments in a real laboratory environment, measuring the received irradiation dose at various locations using a luxometer to demonstrate the completion of the disinfection task. Comparative experiments with other complete coverage path planning algorithms show superior performance in terms of disinfection coverage, dose redundancy, UVC lamp energy consumption, and uniformity.

As part of future work, our goal is to dynamically adjust the speed control of the planner based on the energy dose mapped in the irradiation map. We also plan to study the edge effects of unstructured terrain and adjust the dose application scheme based on the real-time posture of the robot. During the robot’s movement, due to the existence of non-uniform composite light fields, the actual posture of the robot may deviate from the planned posture, resulting in changes in the robot’s irradiation distribution. Due to the limitation of the device’s computational power, periodic correction of the robot’s posture at fixed intervals during the experimental process is necessary to ensure the real-time decision-making and action of the robot. In future work, we will adaptively update the dose application scheme based on the real-time posture of the robot to achieve more accurate irradiation management.

## Figures and Tables

**Table 1:** Parameter settings for the MOPSO algorithm.

Implication	Parameters	Value
Maximum number of iterations	MaxIt	500
Population size	nPop	200
Pareto front size limit	nRep	100
Inertia weight	ω	0.5
Inertia weight decay rate	$w_{damp}$	0.99
Cognitive coefficient	$c_{1}$	0.5
Social coefficient	$c_{2}$	2
Minimum particle velocity	$v_{\max}$	−4
Maximum particle velocity	$v_{min}$	4
$f_{1}$ penalty coefficient	$\omega_{1}$	294
$f_{2}$ penalty coefficient	$\omega_{2}$	0.61
$f_{2}$ penalty coefficient	$\omega_{3}$	70

**Table 2:** Radiation parameters test for single AIGaN-based UVC LED chips.

I (mA)	U (V)	Radiant Flux (mW)	Peak Wavelength (nm)
5	4.700	0.943	272.8
10	4.748	2.016	272.8
20	4.806	4.205	272.7
50	4.903	10.671	272.7
100	4.999	20.880	272.7
200	5.130	39.046	272.8
300	5.232	55.067	272.8
350	5.278	61.301	272.9
500	5.403	77.432	273.1

**Table 3:** Evaluation of disinfection effect.

Scale Factor	Spiral	Voronoi	APF	Ours
Irradiating Trajectory Length (m)	493	37.6	52.6	43.2
Completion Time (s)	3140	2268	2940	1446
Coverage Rate	97.1%	100%	99.2%	100%
Dose Redundancy (mJ/cm^2^)	657.9	288.6	558.4	217.5
Peak-to-Peak Value (mJ/cm^2^)	46.91	28.83	50.01	19.12
Standard Deviation	18.32	8.72	15.63	5.33
Average Navigation Displacement Error	1.2%	2.7%	3.4%	2.2%

**Table 4:** Evaluation of disinfection energy consumption efficiency.

Scale Factor	Spiral	Voronoi	APF	Ours
Irradiating Trajectory Length (m)	493	37.6	152.6	43.2
Movement Duration(s)	3140	188	1940	216
Total Disinfection Duration (s)	3140	2268	1940	1446
Chassis Movement Energy Consumption (kJ)	1808.6	1306.3	1117.4	832.8
Radiation Energy Consumption (kJ)	122.4	73.8	75.6	47.7
Total Disinfection Energy Consumption (kJ)	1931.1	1380.2	1193.1	880.6
Disinfection Electricity Consumption (kWh)	0.54	0.38	0.33	0.25

**Table 5:** The irradiation dose required for UVC inactivation of pathogens [1].

Pathogen Type	Pathogen Name	Inactivation Dose Threshold
Bacteria	Pseudomonas aeruginosa biofilm [1]	7.9 mJ/cm^2^
Virus	Aichi virus [1]	100 mJ/cm^2^
Virus	Bacteriophage MS2 [1]	96 mJ/cm^2^
Virus	Hepatitis A virus [1]	60 mJ/cm^2^
Virus	SARS-CoV-2 [1]	16.9 mJ/cm^2^
Virus	Aerosolized ssRNA virus [1]	7.1 mJ/cm^2^
Virus	Enveloped Influenza A virus (H1N1) [1]	80 mJ/cm^2^
Virus	Feline Calicivirus Influenza A (H5N1) [1]	6 mJ/cm^2^

Data Source [1].

## Data Availability

The data that support the findings of this study are available from the corresponding author upon reasonable request.
